# Biological effects of ^18^F-FDG administration and CT dose during PET/CT: chromosomal aberrations in a two-center prospective observational study

**DOI:** 10.1007/s00330-026-12731-0

**Published:** 2026-07-17

**Authors:** Yuri Kawashima, Yasuha Kinugasa, Mana Ishibashi, Wataru Fukumoto, Yoshihiro Miyata, Reiko Ideguchi, Chiemi Sakai, Mari Ishida, Seiko Hirota, Shinji Yoshinaga, Ikuno Nishibuchi, Yuji Murakami, Gloriamaris Loy-Caraos, Namkhai Bayasgalan, Suvd Bayarjargal, Lin Shi, Jiying Sun, Yasunori Horikoshi, Morihito Okada, Yuko Nakamura, Kazuo Awai, Takashi Kudo, Satoshi Tashiro

**Affiliations:** 1 Research Institute for Radiation Biology and Medicine, Hiroshima University, Hiroshima, Japan; 2 Department of Cellular Biology, Research Institute for Radiation Biology and Medicine, Hiroshima University, Hiroshima, Japan; 3 Medical Infection System Department, Research Institute, Nozaki Tokushukai Hospital, Osaka, Japan; 4 Department of Diagnostic Radiology, Graduate School of Biomedical Health Sciences, Hiroshima University, Hiroshima, Japan; 5 Department of Surgical Oncology, Research Institute for Radiation Biology and Medicine, Hiroshima University, Hiroshima, Japan; 6 Department of Radioisotope Medicine, Atomic Bomb Disease Institute, Nagasaki University, Nagasaki, Japan; 7 Department of Cardiovascular Physiology and Medicine, Graduate School of Biomedical Health Sciences, Hiroshima University, Hiroshima, Japan; 8 Department of Health and Nutrition, Faculty of Health Sciences Hiroshima Shudo University, Hiroshima, Japan; 9 Department of Environmetrics and Biometrics, Research Institute for Radiation Biology and Medicine, Hiroshima University, Hiroshima, Japan; 10 Department of Radiation Oncology, Graduate School of Biomedical and Health Sciences, Hiroshima University, Hiroshima, Japan; 11 Department of Science and Technology, Philippine Nuclear Research Institute, Quezon City, Philippines; 12 Department of Radiology, The Second Affiliated Hospital of Shandong First Medical University, Tai’an, China

**Keywords:** Positron emission tomography computed tomography, Fluorodeoxyglucose F18, Chromosome aberrations, Radiation dosage, Radiation effects

## Abstract

**Objective:**

To assess the radiation effects of internal ^18^F-fluorodeoxyglucose (FDG) administration and external CT exposures during PET/CT, using reduced-dose and standard-dose CT protocols, on chromosomes in peripheral blood lymphocytes (PBLs).

**Materials and methods:**

This prospective two-center observational study included patients undergoing clinically indicated FDG-PET/CT using different CT dose protocols. Peripheral blood was sampled at three time points: before FDG administration, after FDG administration, and after CT acquisition. Unstable chromosomal aberrations (CAs) in PBLs were quantified at each time point. Non-parametric tests were used for group comparisons, and associations between radiation dose parameters and changes in CAs were evaluated using generalized linear mixed-effects models.

**Results:**

A total of 101 consecutive patients were enrolled in the reduced-dose protocol center (Center RD; mean age 70 ± 9 years [standard deviation], 55 men) and 56 patients in the standard-dose protocol center (Center SD; 64 ± 11 years, 31 men). The CA-evaluable population comprised 101 patients in Center RD and 55 patients in Center SD. FDG administration did not significantly increase CAs at either center. Following CT acquisition, no significant increase in CAs was observed with the reduced-dose protocol, whereas CAs increased significantly with the standard-dose protocol (Center SD: *p* = 0.016).

**Conclusion:**

FDG administration had no detectable impact on CAs, whereas CT effective dose was associated with CA frequency after multivariable adjustment, with no evidence of a synergistic interaction with FDG. These findings support CT dose optimization and the use of reduced-dose CT protocols in PET/CT when clinically appropriate.

**Key Points:**

***Question*** *How do internal 18F-fluorodeoxyglucose (FDG) and external CT exposures during PET/CT affect chromosomes, and how do these effects differ between reduced- and standard-dose CT protocols?*

***Findings**** Chromosomal aberrations increased after standard-dose PET/CT, and CT effective dose, but not FDG effective dose, was independently associated with chromosomal aberration frequency after multivariable adjustment*.

***Clinical relevance**** CT effective dose was independently associated with chromosomal aberration frequency, while age and prior chemotherapy also influenced CA frequency. This provides biological justification for CT dose optimization and broader use of reduced-dose PET/CT when clinically appropriate*.

**Graphical Abstract:**

**Figure:**
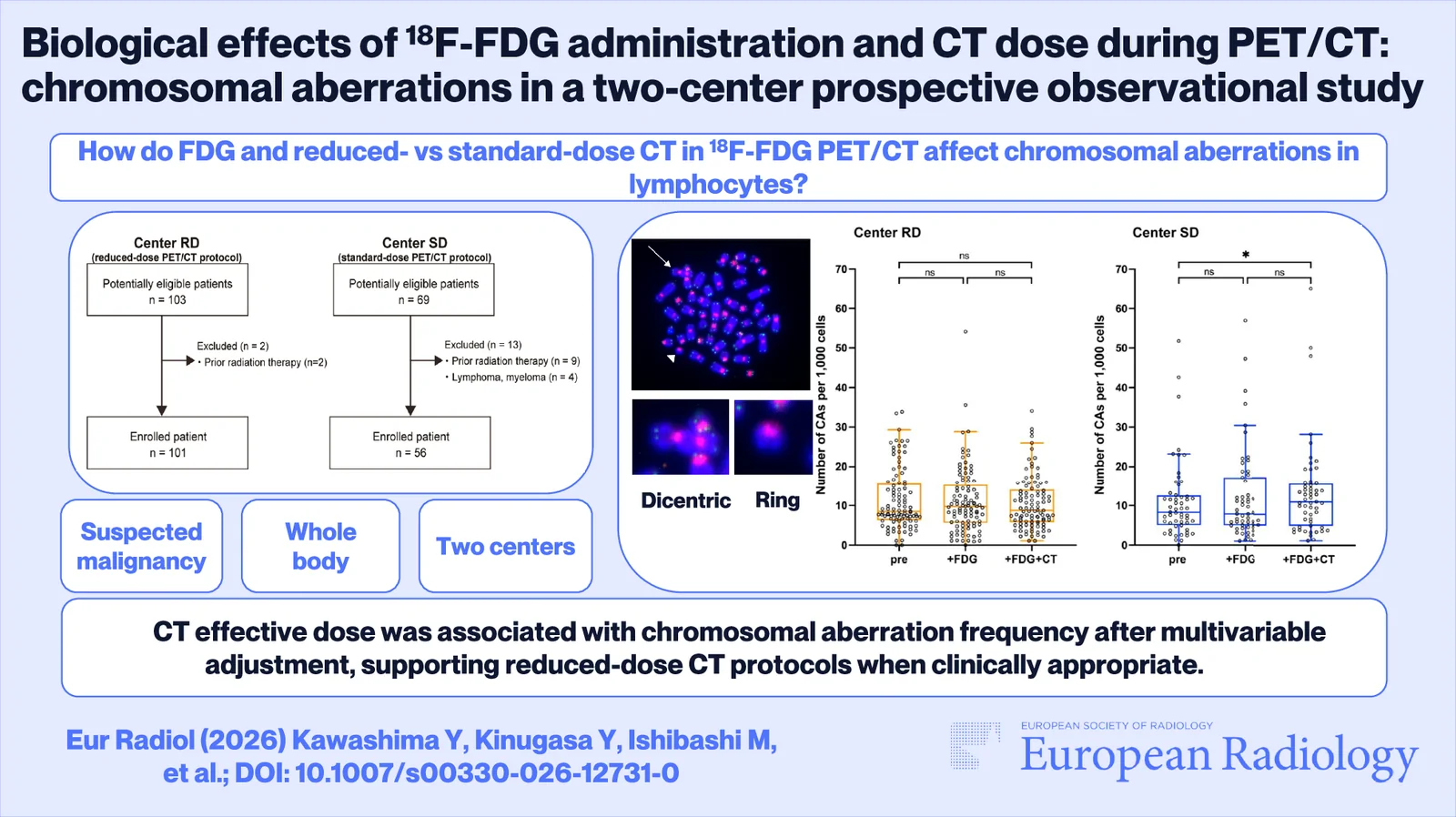

## Introduction

^18^F-fluorodeoxyglucose (FDG)-PET/CT is an indispensable imaging modality, used in approximately 1% of health screening examinations for cancer detection [1–5]. Because PET/CT combines internal exposure from FDG with external exposure from CT, radiation safety and the accurate characterization of its biological effects remain major concerns. While guideline-based recommendations exist for FDG administration [6], there is no clear consensus regarding optimal CT dose setting for PET/CT examinations [6, 7]. Epidemiological evidence suggests that medical imaging may contribute to increased cancer risk [8–13]. Consequently, in accordance with the as low as reasonably achievable (ALARA) principle [14], efforts toward dose reduction, particularly from the CT component, continue to be pursued [15–17]. Nevertheless, real-world clinical data indicate that CT doses in PET/CT are often not reduced, and in many cases are comparable to or exceed those used in diagnostic CT protocols [18]. Biological evidence supporting reduced-dose CT protocols in PET/CT is limited. Existing studies of FDG-related biological effects are small and rely primarily on transient DNA damage markers, such as γ-H2AX [19–24]. Furthermore, contrast-enhanced CT induces more DNA damage compared to non-contrast CT [25], whereas FDG administration did not amplify DNA damage in PET/MRI [23]. However, whether internal FDG exposure and external CT radiation interact synergistically during PET/CT remains unclear. To address these questions, we established a high-sensitivity chromosomal aberration (CA) assay and demonstrated its clinical utility [26–30]. CAs represent the most robust and quantifiable biomarkers of radiation-associated cancer risk [31–34], and their measurement window is sufficiently flexible for clinical studies [35–39].

Building on this platform, we subsequently compared reduced-dose and standard-dose chest CT protocols in a large patient population, demonstrating the biological superiority of the low-dose protocol [28]. These findings support the use of CA analysis as a suitable biomarker for evaluating biological effects in complex radiation exposure settings such as PET/CT.

The present study was designed to address the clinical and biological gaps. Specifically, we aimed (1) to quantitatively distinguish the acute biological contributions of internal FDG exposure and external CT exposure using CA analysis, with assessment of potential interactions between the two exposure modalities; and (2) to define a biologically acceptable CT dose range for PET/CT based on chromosomal aberration data. These results are intended to provide a robust biological foundation for dose-optimization strategies in FDG-PET/CT and to support more accurate communication of radiation-related risks during patient counseling and informed consent.

## Materials and methods

### Study design and setting

This prospective two-center observational study was approved by the Institutional Review Boards of Hiroshima University Hospital (approval No. E2012-0579) and Nagasaki University Hospital (approval No. 18101523), and written informed consent was obtained from all patients in this study. All procedures were carried out in accordance with the Helsinki Declaration and its later amendments. Patients who underwent clinically indicated FDG-PET/CT at two hospitals using different CT dose protocols were prospectively enrolled between April 2019 and January 2022. Hiroshima University Hospital, where a reduced-dose PET/CT protocol is routinely used, was designated as Center RD, and Nagasaki University Hospital, where a standard-dose PET/CT protocol is used, as Center SD.

### Participants

**Fig. 1:**
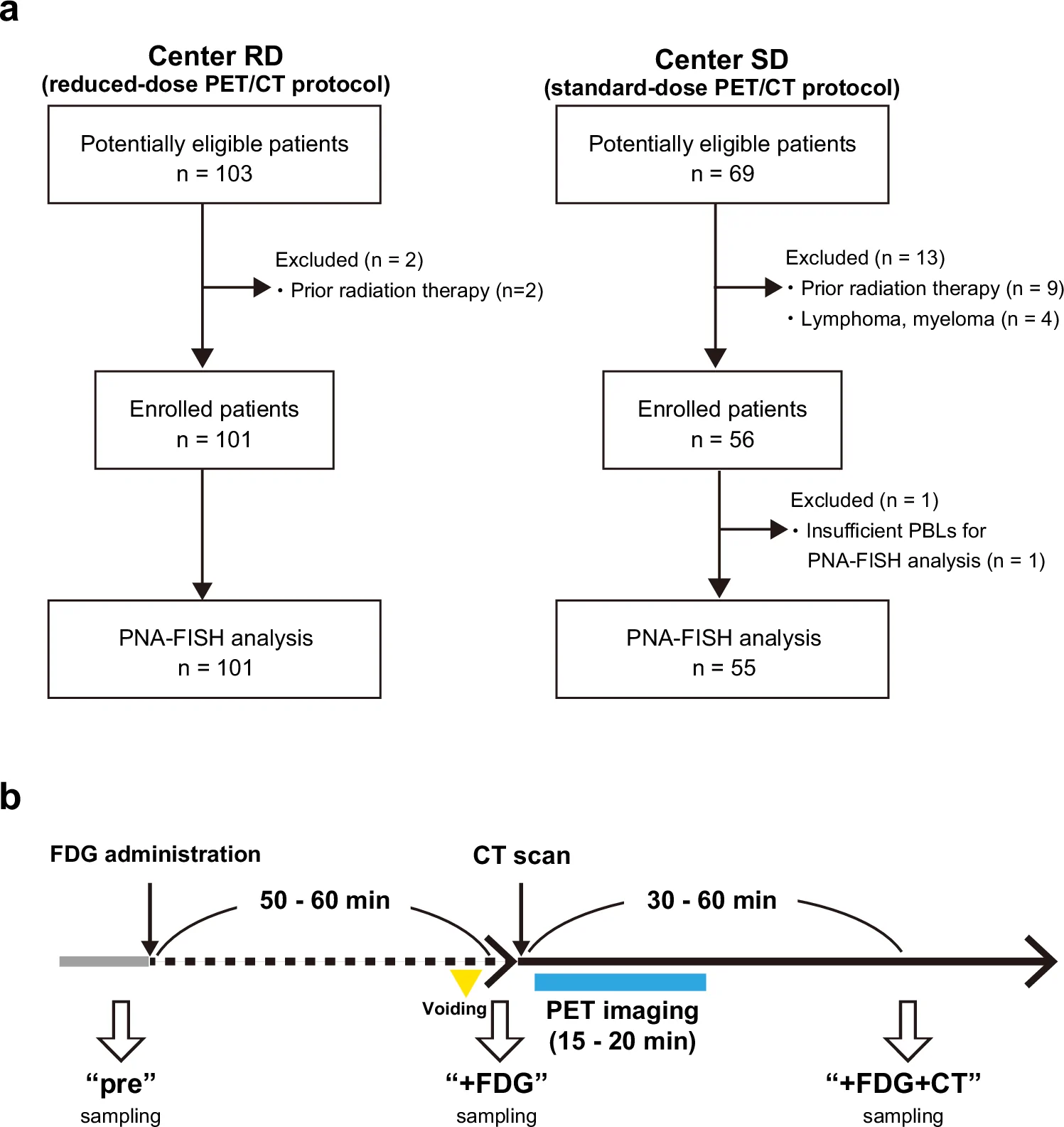
Patient flow and PET/CT blood-sampling schedule. **a** Flowchart of patient enrollment at the two centers. Center RD and Center SD denote the reduced‑dose and standard‑dose PET/CT centers, respectively. Peptide nucleic acid fluorescence in situ hybridization (PNA-FISH) was used to detect chromosomal aberrations (CAs) in peripheral blood lymphocytes (PBLs). Patients included in PNA-FISH analysis were considered CA-evaluable patients. **b** PET/CT procedure and blood‑sampling schedule. Peripheral blood was collected immediately before 18 F‑fluorodeoxyglucose (^18^F‑FDG) administration (“pre”), 50–60 min after FDG administration (“+FDG”), and 30–60 min after CT acquisition (“+FDG + CT”)

A total of 103 consecutive patients at Center RD and 69 at Center SD who underwent clinically indicated FDG-PET/CT were considered potentially eligible (Fig. 1a). Patients with a history of radiation therapy and those with lymphoma and myeloma were excluded. Ultimately, 101 patients at Center RD, most of whom were referred for suspected thoracic malignancy, and 56 patients at Center SD with suspected malignancies at various sites (no restriction on tumor location) were enrolled in this study. At both centers, most of the PET/CT examinations were requested for oncologic evaluation rather than for non-oncologic indications.

### PET/CT acquisition protocol and CT parameters

The whole-body PET/CT scan was performed using Biograph mCT scanners (Siemens Healthineers) with a 64-slice at Center RD or a 16-slice helical CT at Center SD. All patients fasted for at least 4 h before FDG administration and rested quietly for 1 h before the PET/CT acquisition. Blood glucose levels were measured before FDG administration and confirmed to be below 200 mg/dL.

The scanning range extended from the vertex to the mid-thigh of patients. Data acquisition began with a non-contrast CT scan for attenuation correction. CT scan parameters were as follows: tube voltage, 120 kV; Quality ref. mAs 40, 0.5 s; collimation 64 × 0.6 mm; pitch 1.4 for 64-slice helical CT at Center RD. For 16-slice helical CT at Center SD, they were as follows: tube voltage 120 kV; Quality ref. mAs 70, rotation time 0.5 s; collimation 16 × 1.2 mm; pitch 0.8. Anatomy-based tube current modulation (Care Dose 4D™; Siemens Healthineers) was applied in all patients. The dose-length product (DLP) was calculated and recorded for each CT examination. Because DLP is determined by CTDI and scan length, and CTDI reflects scanner configuration and acquisition parameters, DLP is generally regarded as an integrated summary metric of CT radiation exposure across different scanner configurations and imaging protocols.

PET acquisition followed immediately, with emission acquisition times ranging from 90 to 180 s per bed position across a 162-mm axial field of view. PET images were reconstructed with a slice thickness of 6.0 mm using the standard ordered-subset expectation maximization (OSEM) algorithm.

### Blood sampling and chromosomal analysis

**Fig. 2:**
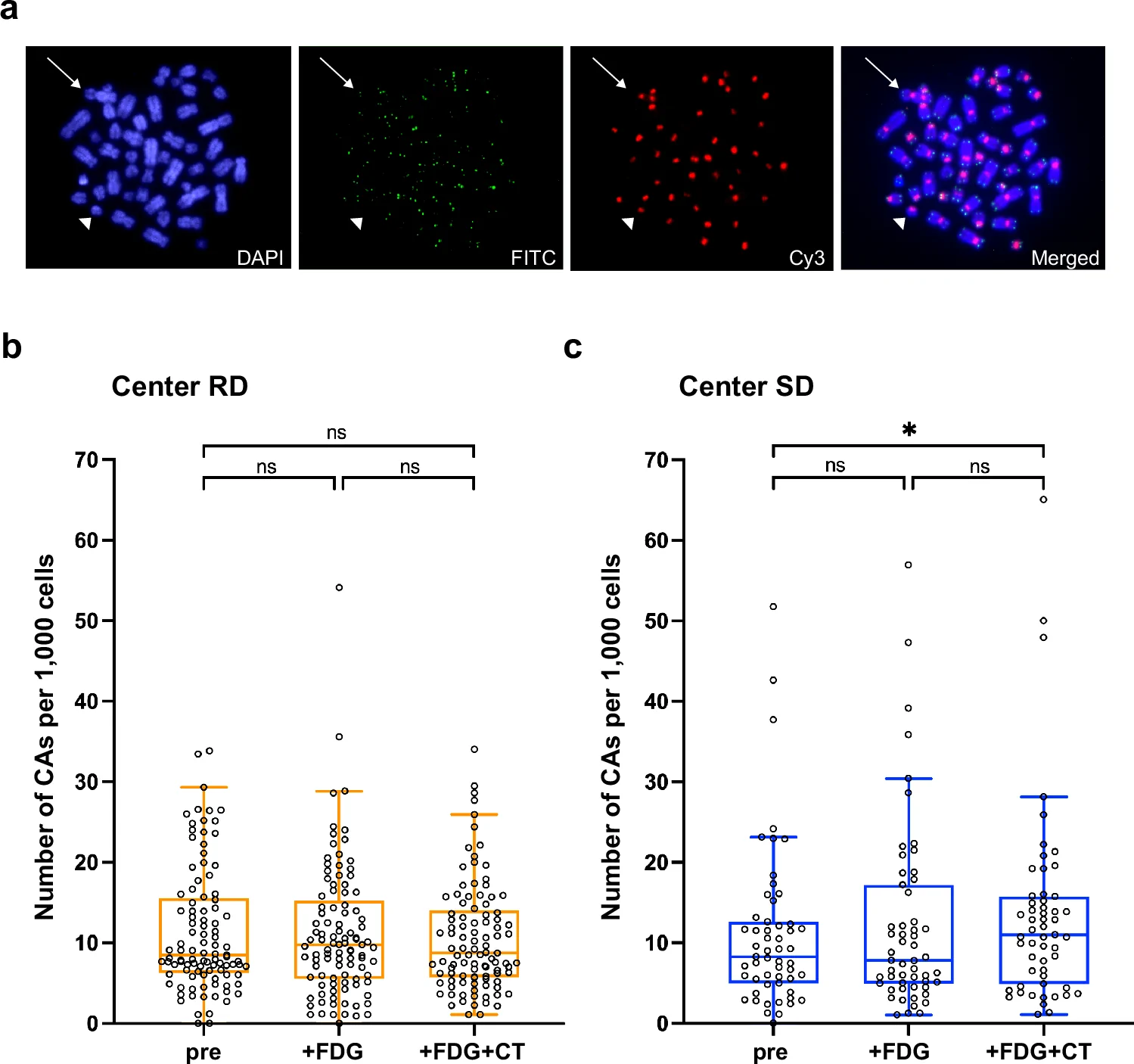
Chromosomal aberrations at each sampling point. **a** Representative metaphase lymphocyte showing chromosomal aberrations. A dicentric chromosome with two centromeres (arrow) and a ring chromosome lacking telomeres (arrowhead) are indicated. Chromosomal DNA is stained with 4′,6-diamidino-2-phenylindole (DAPI) (blue); telomeres with fluorescein isothiocyanate (FITC) (green); centromeres with Cy3 (red); the right panel shows the merged image. **b**, **c** Number of chromosomal aberrations (CAs) per 1000 cells at each sampling point in **b** Center RD (reduced-dose PET/CT protocol) and **c** Center SD (standard-dose PET/CT protocol). Within-center temporal changes were first assessed using the Friedman test. For Center RD, “ns” indicates a non-significant overall Friedman test. For Center SD, pairwise comparisons were performed using Wilcoxon signed-rank tests with Holm adjustment because the Friedman test was significant. Pairwise comparisons: “pre” vs “+FDG,” “+FDG” vs “+FDG + CT,” and “pre” vs “+FDG + CT.” * *p* < 0.05; ns, not significant

Peripheral blood samples (5 mL) were collected at three time points: immediately before FDG administration (“pre”), 50–60 min after FDG administration (“+FDG”), and 30–60 min after CT acquisition (“+FDG + CT”) (Fig. 1b). The 50–60 min post-FDG sampling window was selected in accordance with the EANM guidelines, which recommend a standard uptake interval of 60 min between FDG administration and the start of PET acquisition [7]. As this was an observational study conducted alongside routine clinical care, PET acquisition and subsequent clinical procedures intervened between CT and blood collection, resulting in a post-CT sampling interval of 30–60 min with minor variation across participants. Unstable chromosomal aberrations, particularly dicentric and ring chromosomes in peripheral blood lymphocytes (PBLs), were quantified using peptide nucleic acid fluorescence in situ hybridization (PNA-FISH) in a blinded manner, as previously described [26, 28] (Fig. 2a). CA scoring was performed using a blinded semi-automatic workflow. Metaphase spreads were first automatically searched using the Metafer system, and only analyzable metaphases were selected according to predefined quality criteria; inadequate or uninterpretable metaphases were excluded [40]. For each sample, more than 1000 analyzable metaphases were evaluated, and the same criteria were applied consistently across all sampling time points. Aberrations identified by the software, particularly dicentric and ring chromosomes, were visually confirmed by an experienced technician as an independent verification step. The validity of this semi-automatic approach has been demonstrated previously [28].

### Calculation of committed effective dose fractions after FDG administration

Using the adult committed effective dose coefficient for FDG (0.019 mSv/MBq; ICRP publication 106, Table C.9.4) [41] as a reference, we estimated the fractional contribution of each post-injection time window to the committed effective dose (0–∞). The model was based on the FDG biokinetics and urinary bladder model framework described in ICRP publication 106 Annex C.9, with a physical half-life of FDG T_phys_ = 1.83 h [41]. Voiding was modeled as instantaneous complete emptying of the urinary bladder, reflecting our clinical protocol in which patients were instructed to void immediately before the second blood sampling (“+FDG”).

To assess the impact of variability in blood sampling timing in clinical practice, three representative sampling scenarios (minimum, mean, and maximum) were evaluated independently: (1) 0–50, 50–80, and > 80 min (voiding at 50 min, immediately before the “+FDG” blood sampling); (2) 0–55, 55–100, and > 100 min (voiding at 55 min); and (3) 0–60, 60–120, and > 120 min (voiding at 60 min). In all scenarios, the voiding interval after the first void was assumed to follow the ICRP adult standard of 3.5 h.

The fractional contribution of each time window was calculated by numerically integrating a time-dependent weighting function representing the contribution to the committed effective dose over each interval and dividing the interval-specific area under the curve by the total area under the curve (integration upper limit, 24 h). The results are presented as the time-dependent weighting function and the cumulative committed effective dose fraction curve (Supplementary Fig. S1). Detailed equations and parameter settings are provided in the Supplementary Methods.

### CT dose calculation

The effective dose from the CT scan was calculated by multiplying the DLP (mGy·cm) by a conversion factor of 0.013 mSv/mGy·cm [42]. The CT blood dose for patients at Center RD was estimated using a web-based dosimetry calculator (WAZA-ARI: http://waza-ari.nirs.qst.go.jp) [43, 44], with organ dose weighted by organ blood content values [45] from the International Commission on Radiological Protection (ICRP Publication 89) [46]. The relationship between blood dose and DLP was modeled using linear regression for each sex (man: blood dose = 0.016 × DLP + 0.09, woman: blood dose = 0.019 × DLP − 0.01). For patients at Center SD, where only DLP data were available, blood dose was estimated using these regression equations.

### Statistical analysis

#### Sample size calculation

Sample size calculations for chromosomal aberration endpoints were performed separately for each center. Assuming a paired *t*-test with 80% power and a two-sided *α* of 0.05 to detect mean increases in CA frequency of 0.7 and 2.1 per 1000 cells at Center RD and Center SD, respectively, the required numbers were 95 and 45 patients [28]; our final study population (101 and 56 patients, respectively) exceeded these targets.

#### Descriptive and non-parametric analysis

Normality of continuous variables was assessed using the Shapiro–Wilk test. Normally distributed variables are presented as mean ± standard deviation and were compared using the *t*-test. Non-normally distributed continuous variables, including the number of CAs, are presented as median and interquartile range (IQR) and were compared using the Mann–Whitney *U* test for unpaired data. Within-center temporal changes in chromosomal aberrations across the three sampling time points (“pre,” “+FDG,” “+FDG + CT”) were analyzed using Friedman tests followed by Holm-adjusted Wilcoxon signed-rank tests for pairwise comparisons. Categorical variables were compared using the χ² test. We assessed the association between changes in CAs and each dose parameter using Spearman’s rank correlation coefficient (*ρ*).

#### Multivariable generalized linear mixed-effects models

Associations between dose parameters and changes in unstable CAs were evaluated using generalized linear mixed-effects models (GLMMs) with random intercepts for patients to account for within-subject correlation due to repeated measurements. In the primary analysis, CA counts were modeled assuming a Poisson distribution with a log link, incorporating the log of the total number of chromosomes as an offset to estimate CA rates.

The models included fixed effects for center, age, sex, smoking history (ever vs never), history of chemotherapy (yes vs no), effective dose from FDG, and effective dose from CT, and the FDG-by-CT interaction. These covariates were selected a priori as potential confounders based on clinical relevance. Chemotherapy history was available for all patients, whereas smoking history was missing in 5 CA-evaluable patients at Center SD (Supplementary Table S1). The primary GLMM was therefore performed as a complete-case analysis, including 151 CA-evaluable patients with complete covariate data (Supplementary Table S2). Because chemotherapy history was strongly associated with CA counts, the GLMM was repeated in chemotherapy-naïve patients after excluding 26 patients with a history of chemotherapy (Center RD, *n* = 15; Center SD, *n* = 11) from the primary complete-case analytic set (Supplementary Table S2).

Model fit was assessed by examining overdispersion, zero inflation, convergence, singularity, and simulated residual diagnostics using the Residual Diagnostics for Hierarchical (Multi-Level/Mixed) Regression Models (DHARMa) package in R. Two-sided *p*-values < 0.05 were considered statistically significant. All analyses were performed using R software (version 4.5.1, R Foundation for Statistical Computing).

## Results

### Patient characteristics, FDG and CT doses

A total of 101 patients were prospectively enrolled at Center RD (55 men and 46 women; mean age, 70 ± 9 [standard deviation] years) and 56 patients at Center SD (31 men and 25 women; mean age, 64 ± 11 years) (Fig. 1a). Patient demographics and dose parameters were summarized using all enrolled patients: Center RD, *n* = 101; Center SD, *n* = 56 (Fig. 1a, Supplementary Table S2). Although mean age and blood glucose were significantly higher in Center RD, no significant differences were observed for other baseline characteristics (Table 1). In both centers, PET/CT examinations were performed predominantly for oncologic indications, with underlying malignancies identified in the majority of patients (Center RD, 91.1%; Center SD, 83.9%) (Table 1).

**Table 1:** Baseline characteristics of enrolled participants

	Center RD	Center SD	*p*-value
Number of participants, *n*	101	56	
Sex, men/women, *n*	55/46	31/25	1.00
Age (years)	70 ± 9	64 ± 11	**0.005**
Height (cm)	161 ± 10	162 ± 8	0.73
Weight (kg)	58 (50–66)	57 (52–67)	0.84
BMI (kg/m^2^)	22.3 (20.2–24.9)	22.9 (20.0–24.8)	0.95
Underlying malignancy, *n*/*N* (%)	92/101 (91.1%)	47/56 (83.9%)	0.28
Ever smoker, *n* (%)	55/101 (54.5%)	30/51 (58.8%)	0.73
History of chemotherapy, *n*/*N* (%)	15/101 (14.9%)	13/56 (23.2%)	0.27
Blood test			
WBC	5800 (4895–6613)	5900 (5000–6925)	0.47
Lymphocyte (%)	30.3 (23.6–34.7)	27.2 (20.5–35.2)	0.44
Glucose (mg/dL)	115 (103–125)	102 (94–113)	**< 0.001**
CRE (mg/dL)	0.81 (0.68–0.92)	0.75 (0.69–0.87)	0.40
eGFR (mL/min/1.73m^2^)	66 (57–73)	72 (60–79)	0.10

Data are presented as mean ± SD, median (IQR), or *n*/*N* (%), as appropriate

For categorical variables, *n*/*N* (%) indicates the number of patients with the characteristic divided by the number of patients with available data for that variable in each center

Percentages for smoking history were calculated among patients with available smoking-history data; smoking history was missing in 5 patients at Center SD

*p*-values were calculated using the *t*-test, Mann–Whitney *U* test, and χ² test

Statistically significant values are shown in bold

*Center RD* reduced-dose PET/CT protocol, *Center SD* standard-dose PET/CT protocol, *BMI* body mass index, *WBC* white blood cell count, *CRE* serum creatinine level, *eGFR* estimated glomerular filtration rate

The median FDG activity was 249 MBq (IQR 218–290) at Center RD and 222 MBq (IQR 204–247) at Center SD. The time-resolved dose contribution calculated from the model for the representative mean scenario is presented in Table 2. Results for the other scenarios are provided in Supplementary Table S3. The median CT DLP was 260 mGy·cm (IQR 233–297) at Center RD and 706 mGy·cm (IQR 588–870) at Center SD. Consequently, the total effective dose from PET/CT was significantly higher at Center SD (13.3 mSv [IQR, 12.2–15.6]) than at Center RD (8.3 mSv [IQR, 7.5–8.8], *p* < 0.001) (Table 2).

**Table 2:** FDG and CT dose parameters of enrolled participants

	Center RD	Center SD	*p*-value
FDG PET			
FDG administered activity (MBq)	249 (218–290)	222 (204–247)	< 0.001
Effective dose from FDG administration (mSv)			
0–∞ min	4.7 (4.1–5.5)	4.2 (3.9–4.7)	< 0.001
0–55 min	1.5 (1.3–1.8)	1.3 (1.2–1.5)	< 0.001
55–100 min	0.8 (0.7–1.0)	0.7 (0.7–0.8)	< 0.001
100–∞ min	2.4 (2.1–2.8)	2.1 (2.0–2.4)	< 0.001
CT dose			
Dose-length product (DLP, mGy·cm)	260 (233–297)	706 (588–870)	< 0.001
Effective dose from CT (mSv)	3.4 (3.0–3.9)	9.2 (7.6–11.3)	< 0.001
Calculated blood dose (mGy)	4.7 (4.1–5.1)	11.8 (10.6–15.8)	< 0.001
Total PET/CT dose			
Total effective dose from PET/CT (mSv)	8.3 (7.5–8.8)	13.3 (12.2–15.6)	< 0.001

Data are shown for all enrolled patients: Center RD, *n* = 101; Center SD, *n* = 56

Data are presented as median (IQR)

*p*-values were calculated using the Mann–Whitney *U* test

*Center RD* reduced-dose PET/CT protocol, *Center SD* standard-dose PET/CT protocol

### Temporal changes in chromosomal aberrations induced by PET/CT

The CA endpoint analyses were performed in all CA-evaluable patients: Center RD, *n* = 101; Center SD, *n* = 55, after exclusion of one Center SD patient because of insufficient PBLs for PNA-FISH (Fig. 1a, Supplementary Table S2). As shown in Supplementary Fig. S2, baseline median CA frequencies did not differ significantly between centers (Center RD: 8.5 per 1000 cells [IQR, 6.4–15.4]; Center SD: 8.3 per 1000 cells [IQR, 5.3–12.5], *p* = 0.31; Supplementary Table S4).

Within-center temporal changes in CAs across the three sampling time points were not significant at Center RD (Friedman test, *p* = 0.87), whereas they were significant at Center SD (Friedman test, *p* = 0.023; Fig. 2b, c; Supplementary Table S5). Accordingly, Holm-adjusted pairwise comparisons were performed for Center SD. After FDG administration, ΔCAs (“+FDG” − “pre”) did not significantly increase at Center SD (median ΔCAs, 0.0 [IQR, −3.7 to 5.3], *p* = 0.39; Fig. 2b, c; Supplementary Table S5). For the CT component, ΔCAs (“+FDG + CT” – “+FDG”) also did not significantly increase at Center SD (median ΔCAs, 1.7 [IQR, −2.5 to 5.2], *p* = 0.23). In contrast, ΔCAs (“+FDG + CT” – “pre”) showed a significant increase at Center SD (median ΔCAs, 1.2 [IQR, −1.5 to 6.6], *p* = 0.016).

### Dose-response relationships between FDG-PET/CT dose and chromosomal aberrations

The primary complete-case Poisson GLMM included 151 CA-evaluable patients with complete covariate data: Center RD, *n* = 101; Center SD, *n* = 50 (Table 3, Supplementary Table S2). The model incorporated center, sex, age, smoking history, history of chemotherapy, FDG effective dose, CT effective dose, and the FDG-by-CT interaction. The primary Poisson GLMM showed no evidence of overdispersion or problematic zero inflation, and residual diagnostics did not indicate major model misfit (Supplementary Table S6 and Fig. S3). The analysis demonstrated that center, sex, smoking history, and FDG effective dose were not independently associated with CA counts (Table 3). In contrast, age (*p* = 0.043) and CT effective dose (*p* = 0.008) showed significant associations with CA counts. The strongest effect was observed for history of chemotherapy (*p* < 0.001). These results were consistent with those of the sensitivity model excluding the FDG-by-CT interaction term (Supplementary Table S7).

**Table 3:** Primary complete-case Poisson generalized linear mixed model for factors associated with CA frequency

Primary complete-case GLMM (*n* = 151 patients, 453 observations)				
	Rate ratio	95% CI	*p*-value	
Center				
SD vs RD	0.99	0.81–1.22	0.94	
Sex				
Male vs female	1.07	0.85–1.33	0.58	
Age (per 10 years)	1.10	1.00–1.21	**0.043**	*
Smoking				
Ever vs never	1.13	0.90–1.41	0.29	
Chemotherapy history				
Yes vs no	2.04	1.60–2.59	**< 0.001**	***
FDG effective dose (per 1 mSv)	0.93	0.78–1.10	0.38	
CT effective dose (per 1 mSv)	1.01	1.00–1.02	**0.008**	**
Interaction: FDG effective dose × CT effective dose (per 1 mSv × 1 mSv)	0.95	0.87–1.03	0.23	

Chemotherapy-naïve complete-case GLMM (*n* = 125 patients, 375 observations)				
	Rate ratio	95% CI	*p*-value	
Center				
SD vs RD	0.92	0.73–1.15	0.44	
Sex				
Male vs female	0.96	0.75–1.22	0.73	
Age (per 10 years)	1.11	1.01–1.22	**0.031**	*
Smoking				
Ever vs never	1.30	1.02–1.64	**0.032**	*
FDG effective dose (per 1 mSv)	0.98	0.78–1.24	0.90	
CT effective dose (per 1 mSv)	1.01	1.00–1.02	**0.031**	*
Interaction: FDG effective dose × CT effective dose (per 1 mSv × 1 mSv)	0.98	0.88–1.10	0.77	

The primary GLMM was performed as a complete-case analysis and included 151 CA-evaluable patients: Center RD, *n* = 101; Center SD, *n* = 50

Five CA-evaluable patients at Center SD were excluded because of missing smoking-history data

The chemotherapy-naïve analysis was derived from this complete-case population by excluding patients with a chemotherapy history

Detailed analytic sets are shown in Supplementary Table S2

Rate ratios for age, FDG effective dose, and CT effective dose are expressed per 10-year, 1-mSv, and 1-mSv increase, respectively

Statistically significant values are shown in bold

* *p* < 0.05; ** *p* < 0.01; *** *p* < 0.001

*RD* reduced-dose, *SD* standard-dose, *CI* confidence interval, *CA* chromosomal aberration, *GLMM* generalized linear mixed-effects model

As a sensitivity analysis, we repeated the multivariable analysis using a negative binomial GLMM, and the overall results were materially unchanged (Supplementary Table S8). In the sensitivity analysis restricted to chemotherapy-naïve patients, age and CT effective dose remained statistically significant, while smoking history also reached statistical significance in both the Poisson and negative binomial models (Table 3, Supplementary Table S8).

**Fig. 3:**
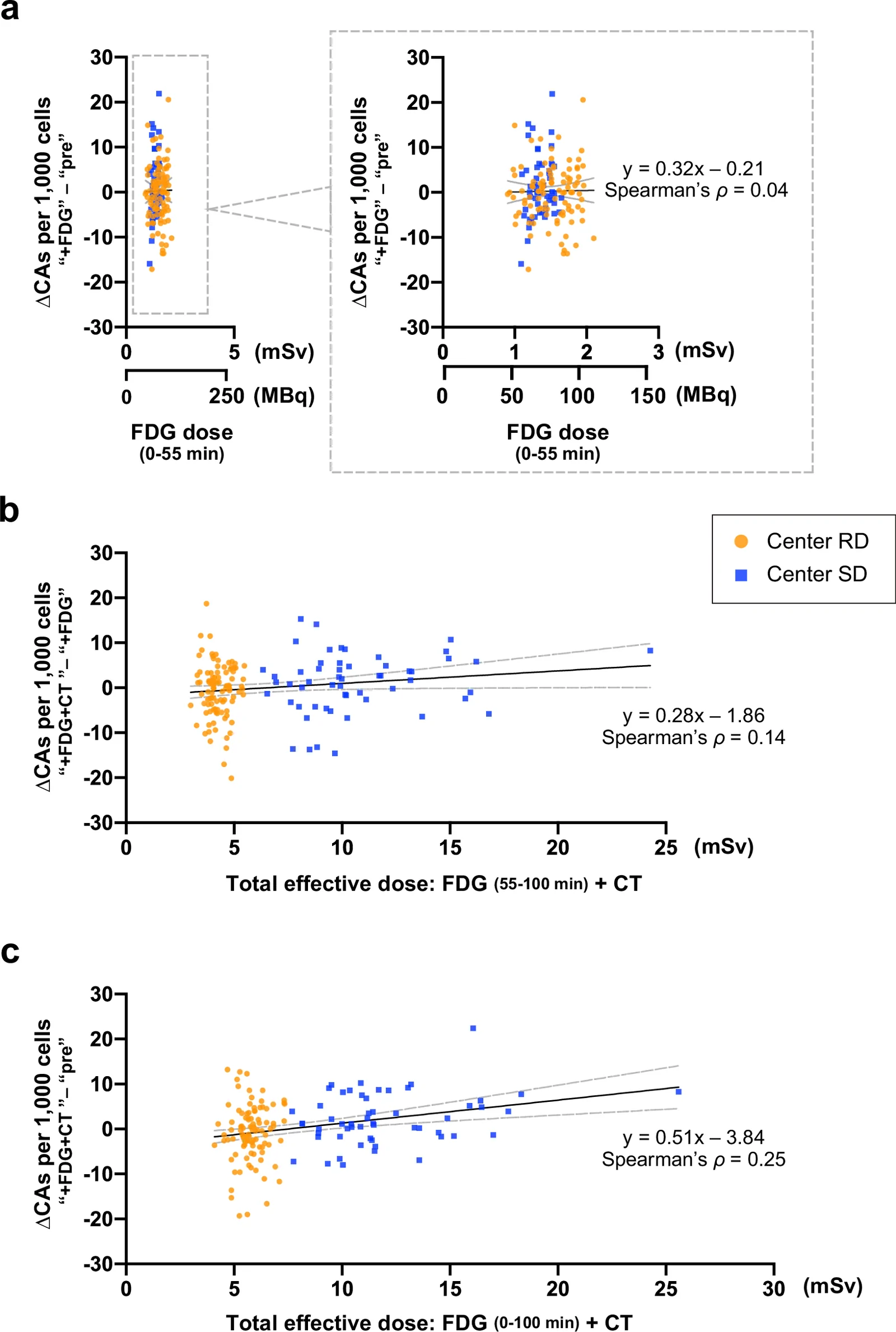
Correlations between FDG, CT and total PET/CT dose and changes in chromosomal aberrations. **a** Correlation between the change in CAs per 1000 cells at “+FDG” versus “pre” (ΔCAs = “+FDG” − “pre”) and FDG effective dose. The *x*-axis shows administered FDG activity (MBq, lower axis) and the corresponding effective dose (mSv, upper axis). The left panel shows the full range, and the right panel shows an enlarged view of the dashed region. **b** Correlation between the change in CAs per 1000 cells at “+FDG + CT” versus “+FDG” (ΔCAs = “+FDG + CT” − “+FDG”) and effective dose from FDG (55–100 min) and CT. The *x*-axis shows effective dose from FDG (55–100 min) and CT (mSv). **c** Correlation between the change in CAs per 1000 cells at “+FDG + CT” versus “pre” (ΔCAs = “+FDG + CT” − “pre”) and total effective dose from PET/CT (FDG (0–100 min) + CT, mSv). Sampling time points (“pre,” “+FDG,” “+FDG + CT”) are defined as in Fig. 1. In all panels, orange circles and blue squares represent Center RD and Center SD, respectively. Solid lines indicate simple linear regression fits and dashed lines the 95% confidence intervals; the regression equation and Spearman’s rank correlation coefficient *ρ* are shown in each panel

Based on these findings, we further evaluated dose–response relationships between dose parameters and ΔCAs using linear regression models. Because the regression coefficients did not differ appreciably across sampling scenarios, the mean scenario was used as the representative condition; results for the other scenarios are provided in Supplementary Tables S9–S11. For these CA endpoint regression analyses, all CA-evaluable patients were included: Center RD, *n* = 101; Center SD, *n* = 55 (Fig. 1a, Supplementary Table S2). No correlation was observed between ΔCAs (“+FDG” – “pre”) and effective dose from FDG administration (Spearman’s *ρ* = 0.04; Fig. 3a; Supplementary Table S9). In contrast, a positive correlation was observed between ΔCAs (“+FDG + CT” – “+FDG”) and effective dose from FDG (55–100 min) and CT (Spearman’s *ρ* = 0.14; Fig. 3b; Supplementary Table S10). Notably, the total effective dose from PET/CT (FDG: 0–100 min + CT) showed a positive but modest association with the changes in CAs (“+FDG + CT” – “pre”) (Spearman’s *ρ* = 0.25; Fig. 3c; Supplementary Table S11).

### Subgroup analysis by chemotherapy history

**Fig. 4:**
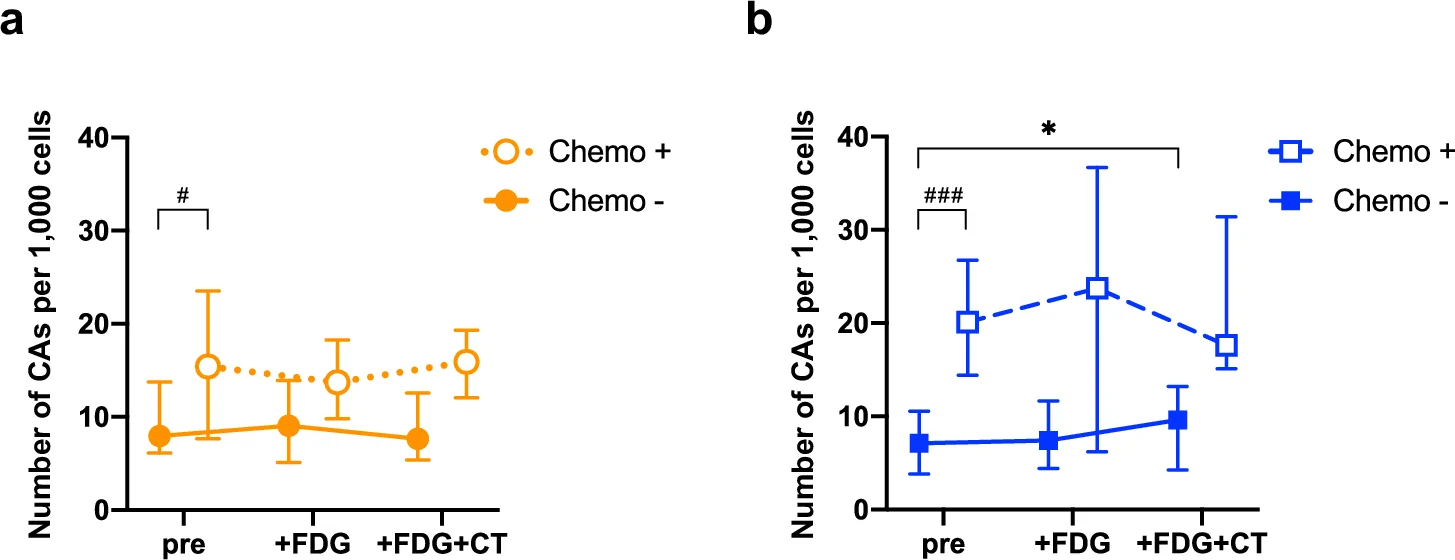
Subgroup analysis according to history of chemotherapy. **a** Center RD; **b** Center SD. In each center, patients were stratified into those with a history of chemotherapy (Chemo +) and those without (Chemo −). Open symbols (circles in **a**, squares in **b**) represent Chemo +, and filled symbols represent Chemo − . The *y*-axis shows the number of chromosomal aberrations (CAs) per 1000 cells. Sampling time points (“pre,” “+FDG,” “+FDG + CT”) are defined as in Fig. 1. Between-group comparisons at baseline (“pre”) within each center were performed with the Mann–Whitney *U* test; # indicates *p* < 0.05, ### indicates *p* < 0.001. Within-group changes over time were assessed using the Friedman test, followed by Wilcoxon signed-rank tests for pairwise comparisons with Holm correction. Pairwise comparisons were performed between “pre” and “+FDG,” “+FDG” and “+FDG + CT,” and “pre” and “+FDG + CT.” * *p* < 0.05; comparisons without an asterisk were not statistically significant after adjustment

Because the history of chemotherapy showed the strongest association with CA frequencies in the multivariable analysis, subgroup analyses were performed. Subgroup analyses by chemotherapy history were performed using the same primary complete-case analytic set as the GLMM (Supplementary Table S2). At both centers, patients with a history of chemotherapy exhibited significantly higher baseline (“pre”) CA counts than chemotherapy-naïve patients (Center RD: *p* = 0.028; Center SD: *p* < 0.001; Fig. 4a; Supplementary Table S12).

Within-center time-course analyses showed no significant temporal changes in CAs at Center RD, irrespective of chemotherapy history. At Center SD, temporal changes were significant only among chemotherapy-naïve patients (Friedman test, *p* = 0.037), whereas no significant temporal changes were observed among patients with a history of chemotherapy. In Holm-adjusted pairwise comparisons among chemotherapy-naïve patients at Center SD, ∆CAs (“+FDG + CT” – “pre”) showed a significant increase (median ΔCAs, 1.2 [IQR, −0.9 to 5.8], *p* = 0.017; Fig. 4b; Supplementary Table S13).

## Discussion

In this two-center prospective observational study (Center RD, *n* = 101; Center SD, *n* = 56), serial blood sampling at three time points (“pre,” “+FDG,” and “+FDG + CT”) enabled separation of the biological effects of FDG administration from CT exposure using CA analysis. Using this approach, we found no measurable contribution of FDG administration to CA formation in peripheral blood lymphocytes, whereas CT-derived radiation dose was suggested to play a larger role in PET/CT-induced CAs. These findings directly address the primary objective of the study and provide biologically grounded evidence relevant to dose optimization in PET/CT acquisition.

First, our findings suggest that CT-related radiation, rather than FDG activity, contributes more strongly to PET/CT-related CA changes. This interpretation was supported by the increase in CAs observed after standard-dose PET/CT and by the positive dose–response relationship with CT dose. At the same time, patient-related factors, particularly age and prior chemotherapy, were also important determinants of CA frequency, with chemotherapy history showing the strongest association. These findings suggest that CT-derived radiation contributes to PET/CT-related CA changes, while the magnitude and detectability of these changes are influenced by baseline patient-related factors. The absence of significant interaction between FDG effective dose and CT effective dose addresses previous theoretical concerns that radiopharmaceutical uptake may potentiate DNA damage [25] and is consistent with reports demonstrating no synergistic biological effects between FDG administration and MRI [23]. CAs are robust biodosimetric endpoints recommended in international guidelines [47–49] and provide a more stable and quantitative assessment than transient DNA damage markers such as γH2AX [19–24]. The observed differences in CA frequency were small (approximately 1–2 per 1000 cells) and within the expected range of biological variability, suggesting limited relevance at the population level. Nevertheless, CA-based assessment may serve as a sensitive tool for evaluating biological effects associated with reduced-dose internal radiation exposure from emerging PET tracers and radiotheranostic agents [50–54].

The second major finding is that CT protocol selection substantially influenced biological effects. FDG activities at both centers were within the ranges recommended by European Association of Nuclear Medicine (EANM) guidelines [7], whereas CT doses differed markedly: Center RD used reduced-dose CT for attenuation correction and anatomical localization, while Center SD applied standard diagnostic CT in accordance with Japanese diagnostic reference levels [55]. In the context of the linear no-threshold (LNT) model and the increasing clinical use of PET/CT, even modest reductions in radiation exposure may be relevant [56]. These findings provide biological support for the ALARA principle [14] and suggest that standard-dose CT during PET/CT should be reserved for clearly justified diagnostic indications.

CA-based biodosimetry further enabled characterization of individual variability in dose response. Patients with a history of chemotherapy exhibited higher and more heterogeneous baseline CA levels, indicating that radiation-induced changes from reduced-dose PET/CT exposure are likely to fall within background variability. In contrast, chemotherapy-naïve patients demonstrated significant radiation-induced increases in CAs following standard-dose PET/CT exposure. Considering the relatively low cancer detection rate (around 1%) in FDG-PET/CT screening examinations [3], it is important to reduce the risk of radiation exposure during such examinations. Our data indicate that a CT dose reduction protocol would be effective in reducing the risk of radiation effects associated with FDG-PET/CT cancer screening. Together, these observations support broader consideration of reduced-dose CT protocols, particularly for screening examinations and for individuals without prior therapeutic exposures, and underscore the value of CA-based metrics for patient-centered risk communication [57, 58].

This study has several limitations. First, patient characteristics differed between centers; although hematologic malignancies were excluded and disease-related effects on baseline CA levels appeared limited, residual confounding cannot be excluded [59, 60]. In addition, although DLP was used as an integrated summary metric of CT exposure, per-organ biological doses may not be identical between the 64-slice and 16-slice CT platforms even when DLP is comparable; this residual scanner-related difference remains a limitation of the between-center comparison. Second, the broad definition of chemotherapy history encompassed heterogeneous treatment modalities, including cytotoxic agents, targeted therapies, and immune checkpoint inhibitors, precluding assessment of drug class–specific effects on CAs. Third, blood sampling time points varied modestly among patients, reflecting real-world clinical practice rather than controlled experimental conditions. In addition, precise estimation of individual FDG administered activity was limited by inter-patient variability in pharmacokinetics and clearance [61, 62]. Fourth, CA analysis was restricted to circulating lymphocytes and did not assess organ-specific radiation effects. Although previous studies have demonstrated that unstable CAs in circulating lymphocytes are detectable in peripheral blood as early as 15 min after chest CT [27, 28], the post-exposure blood sampling in the present study was limited to 50–60 min after FDG administration and 30–60 min after CT acquisition. Consequently, delayed FDG-related genotoxic effects or effects occurring in non-circulating compartments may not have been captured. Therefore, the present findings should be interpreted as indicating the absence of a measurable FDG-related increase in chromosomal aberrations in circulating lymphocytes under these specific experimental conditions.

In conclusion, under the conditions of this study, FDG administration was not associated with a measurable increase in CAs in circulating PBLs during PET/CT imaging, whereas CT-derived radiation dose showed a stronger association with the biological effects observed in this circulating population. These findings provide biological evidence supporting the use of reduced-dose CT protocols from a radiation safety perspective. CA-based biodosimetry offers a robust framework for evaluating biological effects of emerging nuclear imaging and radiotheranostics. These findings may contribute to precision radiation medicine and patient-centered risk communication.

## Supplementary information

Supplementary material

## Abbreviations

ALARA: As low as reasonably achievable
CA: Chromosomal aberration
CT: Computed tomography
DLP: Dose-length product
EANM: European Association of Nuclear Medicine
FDG: ^18^F-fluorodeoxyglucose
GLMM: Generalized linear mixed-effects model
ICRP: International Commission on Radiological Protection
IQR: Interquartile range
PBL: Peripheral blood lymphocyte
PET/CT: Positron emission tomography/computed tomography
PET: Positron emission tomography
PNA-FISH: Peptide nucleic acid fluorescence in situ hybridization
