# Supplementary material for: Biological effects of 18F-FDG administration and CT dose during PET/CT: chromosomal aberrations in a two-center prospective observational study
Source: Eur Radiol. 2026 Jul 17;36(11):8264–76. doi: 10.1007/s00330-026-12731-0 (PMC13661646; doi:10.1007/s00330-026-12731-0)
Supplement: Supplementary file 1 [file 330_2026_12731_MOESM1_ESM.pdf]

Supplementary Materials for

“Biological effects of 18F-FDG administration and CT dose during PET/CT: chromosomal aberrations in a two-center prospective observational study”

Supplementary Methods

Calculation of time-window-specific fractions of the committed effective dose after FDG administration

Supplementary Figures: S1-S3

Supplementary Fig. S1 Estimated fractional contributions of post-injection time windows to the committed effective dose after FDG administration

Supplementary Fig. S2 Comparison of baseline chromosomal aberrations between centers

Supplementary Fig. S3 Residual diagnostics for the fitted model based on simulated residuals

Supplementary Tables: S1-S13

Supplementary Table S1. Missing-data summary for smoking history among CA-evaluable patients

Supplementary Table S2. Patient numbers and analytic sets used in CA endpoint and GLMM analyses

Supplementary Table S3. Distribution of time-window-based effective dose from FDG-PET/CT used in regression analyses, by study center

Supplementary Table S4. Median and interquartile range (IQR) of CAs at each sampling point

Supplementary Table S5. Median and IQR of ΔCAs per 1,000 cells between sampling points

Supplementary Table S6. Model fit diagnostics for the primary Poisson generalized linear mixed model

Supplementary Table S7. Sensitivity Poisson generalized linear mixed model excluding the interaction term between FDG effective dose and CT effective dose

Supplementary Table S8. Sensitivity negative binomial generalized linear mixed model for factors associated with CA frequency

Supplementary Table S9. Association of time-window-based FDG dose calculations with ΔCAs per 1,000 cells (“+FDG” − “pre”)

Supplementary Table S10. Association of time-window-based FDG dose calculations (FDG+CT) with ΔCAs per 1,000 cells (“+FDG+CT” − “+FDG”)

Supplementary Table S11. Association of time-window-based FDG dose calculations (FDG+CT) with ΔCAs per 1,000 cells (“+FDG+CT” − “pre”)

Supplementary Table S12. Median values and IQR for each group in each center

Supplementary Table S13. Median and IQR of ΔCAs for each group in each center

Supplementary Methods

Calculation of time-window-specific fractions of the committed effective dose after FDG administration

Time-window-specific fractions of the adult committed effective dose after ^{18}F-FDG administration were estimated using a reduced time-dependent weighting function derived from the FDG biokinetic model in ICRP Publication 106 Annex C.9 and the adult absorbed dose/effective dose coefficients in Table C.9.4. Let A_0denote the administered activity and $t$the time after injection in hours. The physical half-life of ^{18}F was $T_{\mathrm{phys}} = 1.83$h, and the physical decay constant was $\lambda_p = ln\ (2)/T_{\mathrm{phys}}$. Following the FDG biokinetic model, the initial fraction assigned to "other organs and tissues" was $F_{\mathrm{oth}} = 0.80$. Within this compartment, the retained fraction was $g_{\mathrm{ret}} = 0.70$, whereas the urinary excretion fraction was $f_{\mathrm{ex}} = 0.30$, partitioned into a fast component $g_{\mathrm{fast}} = 0.25$with biological half-time $T_{\mathrm{fast}} = 0.20$ h and a slow component $g_{\mathrm{slow}} = 0.75$with biological half-time $T_{\mathrm{slow}} = 1.5$ h. The corresponding biological rate constants were $\lambda_{\mathrm{fast}} = ln\ (2)/T_{\mathrm{fast}}$and $\lambda_{\mathrm{slow}} = ln\ (2)/T_{\mathrm{slow}}$.

The resulting fractions of administered activity were $b_{\mathrm{ret}} = F_{\mathrm{oth}} g_{\mathrm{ret}} = 0.56$, $b_{\mathrm{fast}} = F_{\mathrm{oth}} f_{\mathrm{ex}} g_{\mathrm{fast}} = 0.06$, and $b_{\mathrm{slow}} = F_{\mathrm{oth}} f_{\mathrm{ex}} g_{\mathrm{slow}} = 0.18$. The normalized activity outside the bladder, $A_{\mathrm{oth}}(t)$, was defined as

$$A_{oth}(t) = b_{ret} e^{-\lambda_p t} + b_{fast} e^{-(\lambda_p + \lambda_{fast})t} + b_{slow} e^{-(\lambda_p + \lambda_{slow})t},$$

where $A_{\mathrm{oth}}(t)$is dimensionless because all activities were normalized by A_0.

Urinary bladder emptying was modeled as instantaneous complete voiding. Let v_0 denote the first voiding time (50/60, 55/60, or 60/60 h, depending on the scenario), and let $v_n = v_0 + 3.5n$ h $(n = 0,1,2, \dots)$denote subsequent voiding times. For a given time t, the most recent voiding time was $t_v = \max\ (\{0\} \cup \{v_n : v_n \leq t\})$. The normalized bladder-contents activity, $B(t)$, was then defined as

$$B(t) = b_{fast}\left[e^{-\lambda_p t} e^{-\lambda_{fast} t_v} - e^{-(\lambda_p + \lambda_{fast})t}\right] + b_{slow}\left[e^{-\lambda_p t} e^{-\lambda_{slow} t_v} - e^{-(\lambda_p + \lambda_{slow})t}\right].$$

By definition, $B(t) = 0$ immediately after each void.

To convert the biokinetic functions into a reduced representation of effective-dose contribution, the ICRP tissue weighting factor for target tissue T was denoted w_T^{ICRP}, and the adult absorbed dose coefficient for the same tissue was denoted d_T(mGy/MBq). For gonads, d_{gonads} was defined as the arithmetic mean of the ovarian and testicular coefficients, consistent with the ICRP effective-dose calculation. We defined $c_T = w_T^{\mathrm{ICRP}} d_T$ and $E_{\mathrm{ref}} = \sum_T c_T$, where $E_{\mathrm{ref}} \approx 0.019$ mSv/MBq. To avoid confusion with w_T^{ICRP}, the three reduced-model coefficients were denoted q_{bl}, q_{phys}, and q_{oth}, where

$$q_{bl} = \frac{c_{bladder}}{E_{ref}},\ q_{phys} = \frac{(c_{lung} + c_{liver})}{E_{ref}},\ q_{oth} = 1 - q_{bl} - q_{phys}.$$

In this reduced model, the physically retained component was represented by the combined lung and liver contribution, whereas the remaining weighted tissues were approximated by $A_{\mathrm{oth}}(t)$. Numerically, $q_{\mathrm{bl}} = 0.3425$, $q_{\mathrm{phys}} = 0.1818$, and $q_{\mathrm{oth}} = 0.4757$.

The time-dependent weighting function, $r(t)$, representing the relative instantaneous contribution to the adult committed effective dose was defined as

$$r(t) = q_{bl}B(t) + q_{phys}e^{-\lambda_p t} + q_{oth}A_{oth}(t).$$

Because only relative time-window fractions were required, $r(t)$ was used as a normalized weighting function, and any common proportionality constant cancels in the final fraction calculation. This reduced function was used only to partition the published adult effective dose coefficient into time intervals and was not used to derive new absolute organ dose estimates.
For a time window $[t_1, t_2]$, the fraction contribution was calculated as

$$P(t_1, t_2) = \frac{\int_{t_1}^{t_2} r(t)dt}{\int_0^{t_{max}} r(t)dt},$$

and the cumulative fraction curve was calculated as

$$F(t) = \frac{\int_0^t r(u)du}{\int_0^{t_{max}} r(u)du}.$$

Numerical integration was performed in R by discrete summation on a uniform time grid with step size $\Delta t = 1/600$h (6 s), using 24 h as the common upper limit for total AUC calculation. The three timing scenarios were evaluated independently as 0–50, 50–80, and >80 min with voiding at 50 min; 0–55, 55–100, and >100 min with voiding at 55 min; and 0–60, 60–120, and >120 min with voiding at 60 min. In these labels, the final interval (>80, >100, or >120 min) denotes the interval from that time point to the common upper integration limit of 24 h. The normalized weighting function $r(t)$ and the cumulative fraction curve $F(t)$ are shown in Supplementary Figure S1.

Reference

International Commission on Radiological Protection (2008) Radiation dose to patients from radiopharmaceuticals. Addendum 3 to ICRP Publication 53. ICRP Publication 106. Ann ICRP 38(1–2):1–197.

Supplementary Figures

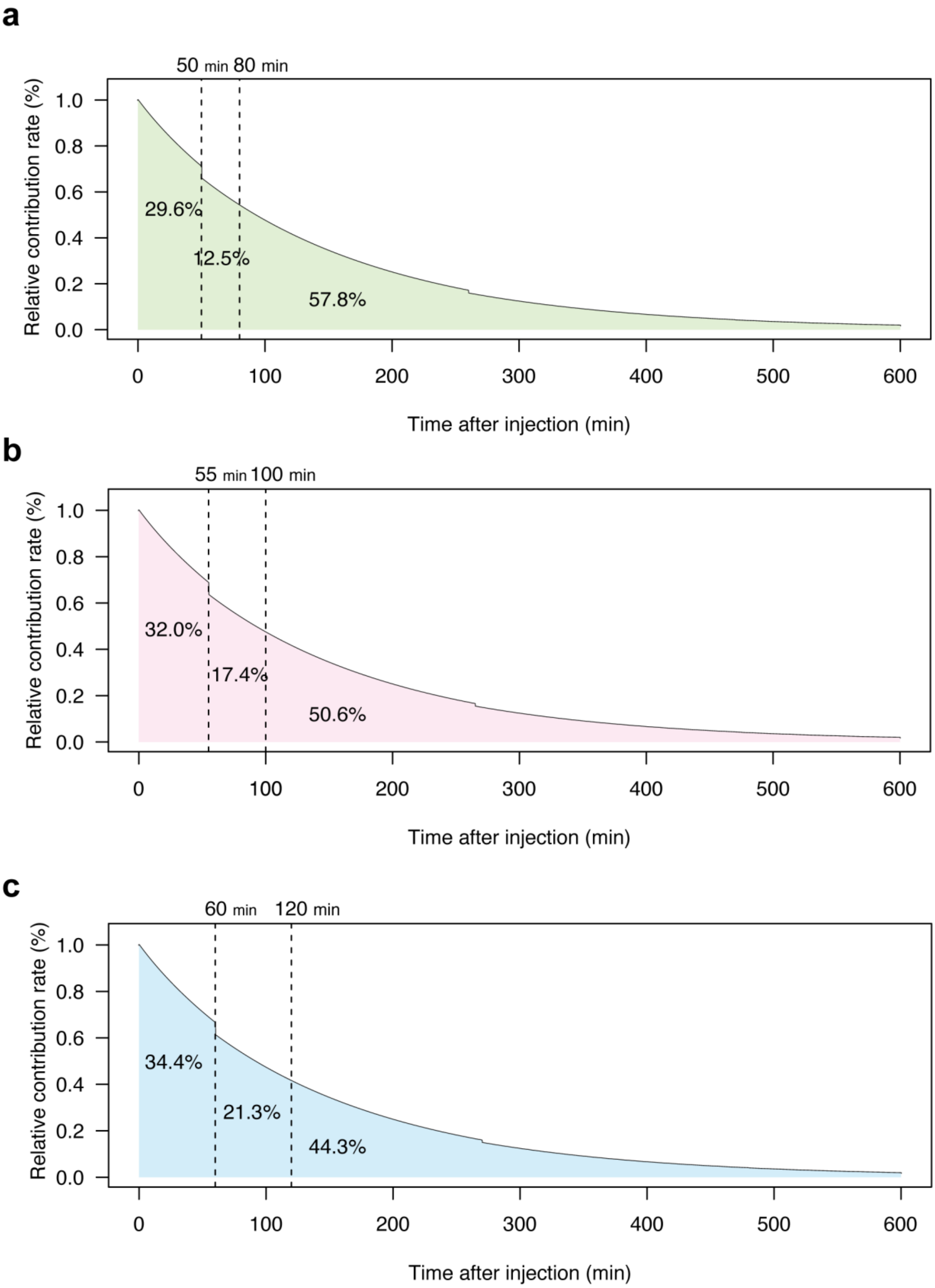

Supplementary Fig. S1 Estimated fractional contributions of post-injection time windows to the committed effective dose after FDG administration

Three representative sampling scenarios were evaluated to reflect the minimum **a**, mean **b**, and maximum **c** timing observed in clinical practice: **a** 0–50, 50–80, and >80 min; **b** 0–55, 55–100, and >100 min; and **c** 0–60, 60–120, and >120 min. Curves show the relative contribution rate over time, and shaded regions indicate the fraction attributable to each time window. Values shown in the panels indicate the corresponding percentages.

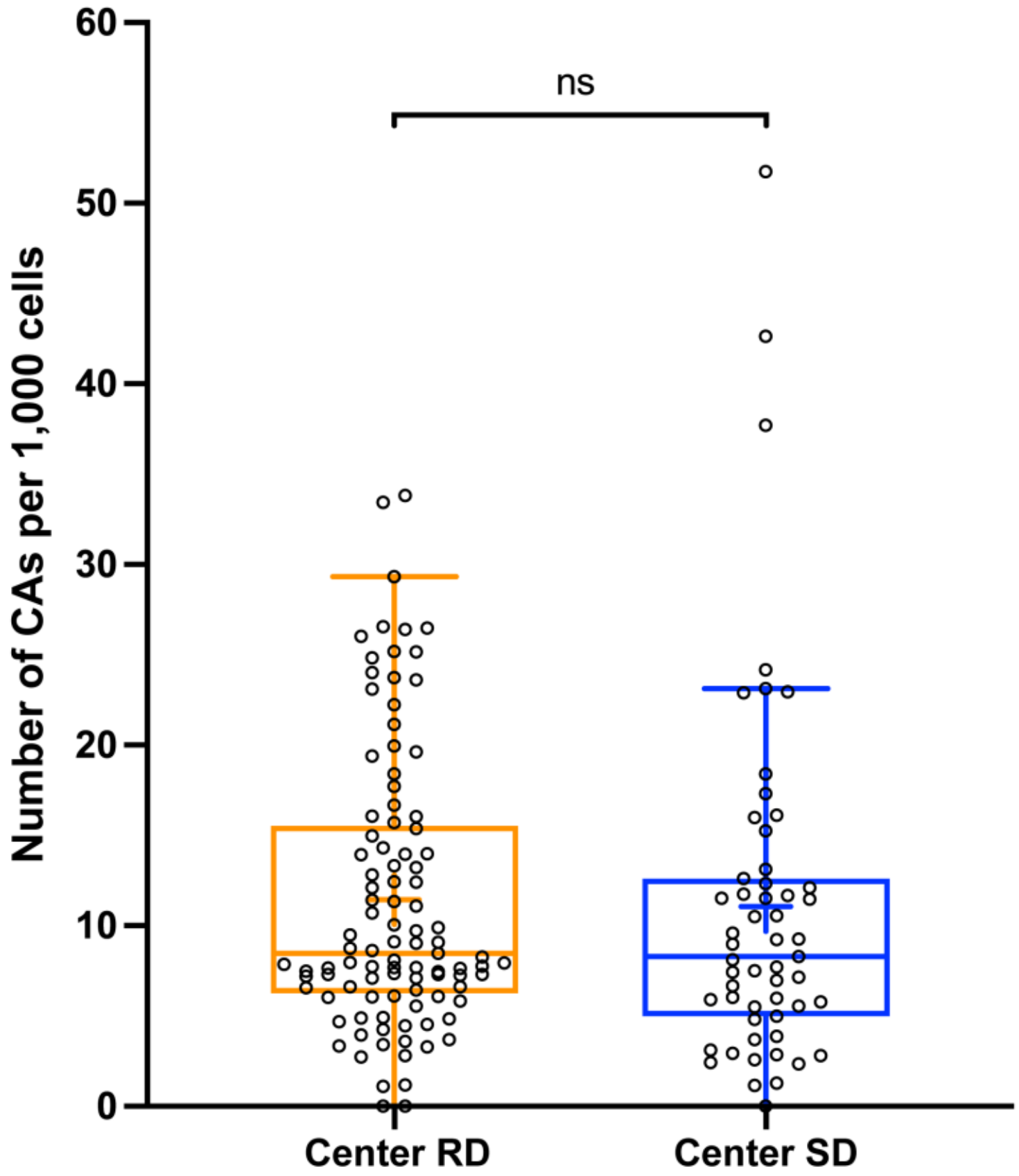

Supplementary Fig. S2 Comparison of baseline chromosomal aberrations between centers
Baseline (“pre”) values between Center RD and Center SD were compared using the Mann–Whitney *U* test ($p = 0.31$). The y-axis shows the number of CAs per 1,000 cells at baseline; “ns” indicates a non-significant difference ($p \geq 0.05$).

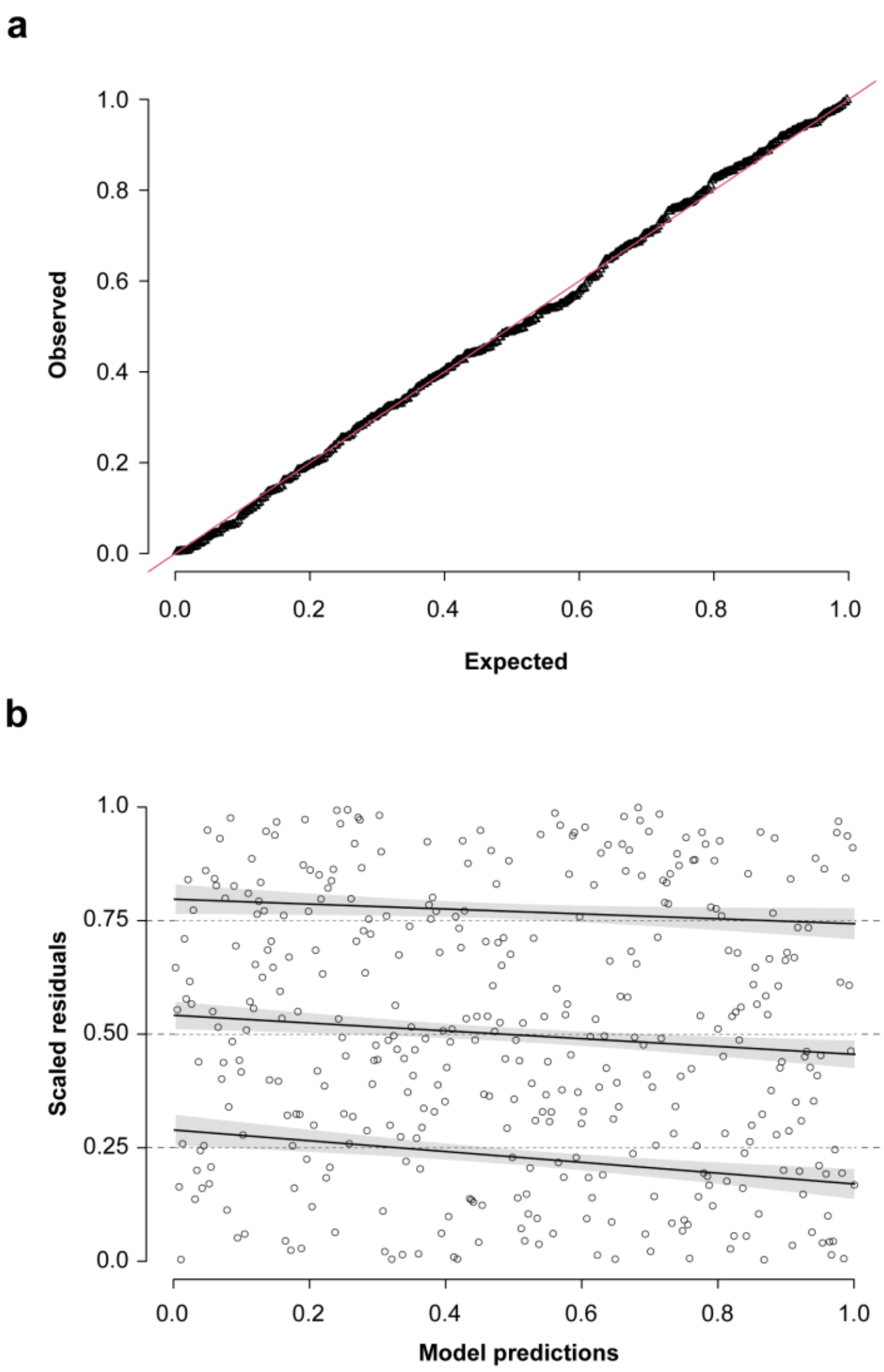

Supplementary Fig. S3 Residual diagnostics for the fitted model based on simulated residuals. Simulated residuals were generated using Residual Diagnostics for Hierarchical (Multi-Level / Mixed) Regression Models (DHARMa), an R package that provides simulation-based residual diagnostics for hierarchical regression models.

a. Quantile–quantile plot of simulated residuals against the expected uniform distribution. The red line indicates the 1:1 expectation. The Kolmogorov–Smirnov test ($p = 0.89$), dispersion test ($p = 0.83$), and outlier test ($p = 1.00$) showed no significant deviation.

b. Simulated residuals plotted against rank-transformed model predictions. Open circles represent scaled residuals, solid black lines indicate quantile regression fits (0.25, 0.50, and 0.75 quantiles), grey shaded areas indicate 95% confidence intervals, and dashed horizontal lines indicate the expected quantiles.

Supplementary Tables

Supplementary Table S1. Missing-data summary for smoking history among CA-evaluable patients

Smoking history of enrolled patients				
	Ever smoker	Never smoker	Missing	Total
Center RD, n	55	46	0	101
Center SD, n	30	21	5	56
Smoking history of CA-evaluable patients				
Center RD, n	55	46	0	101
Center SD, n	29	21	5	55

CA: chromosomal aberration; RD: reduced-dose PET/CT protocol; SD: standard-dose PET/CT protocol.

At Center SD, smoking history was missing in 5 of 56 enrolled patients and in 5 of 55 CA-evaluable patients.

Supplementary Table S2. Patient numbers and analytic sets used in CA endpoint and GLMM analyses

Analysis	Center RD, n	Center SD, n	Included patients, n	Observations, n	Exclusion/selection criteria	Used in
Enrolled patients	101	56	157	–	All enrolled patients	Table 1–2, Table S3
CA-evaluable patients	101	55	156	468	Excluded: n = 1 of 56 enrolled patients at Center SD because of insufficient PBLs for PNA-FISH	Figure 2–4, Table S4–S5, S9–11
Primary GLMM	101	50	151	453	Excluded: n = 5 of 55 CA-evaluable patients at Center SD because of missing smoking-history data	Table 3, Table S7–S8
Chemotherapy-naïve primary GLMM	86	39	125	375	Excluded: n = 26 patients with chemotherapy history from the primary GLMM analytic set (Center RD, n = 15 of 101; Center SD, n = 11 of 50)	Table 3, Table S8

RD: reduced-dose PET/CT protocol; SD: standard-dose PET/CT protocol; CA: chromosomal aberration; GLMM: generalized linear mixed-effects model; PBL: peripheral blood lymphocyte; PNA-FISH: peptide nucleic acid fluorescence in situ hybridization.

Observations indicate repeated measurements across the three blood-sampling time points.

At Center SD, 13 enrolled patients had a history of chemotherapy; however, one was excluded from CA endpoint analyses because of insufficient PBLs for PNA-FISH, and one was excluded from the primary GLMM because of missing smoking-history data.

Thus, 11 Center SD patients with chemotherapy history were excluded when deriving the chemotherapy-naïve primary GLMM analytic set.

Supplementary Table S3. Distribution of time-window-based effective dose from FDG-PET/CT used in regression analyses, by study center

Dose component	Integration time window	Center RD Median (IQR)	Center SD Median (IQR)
Effective dose from FDG PET (mSv)			
Total effective dose	0–∞ min	4.7 (4.1–5.5)	4.2 (3.9–4.7)
Minimum blood sampling time	0–50 min	1.4 (1.2–1.6)	1.2 (1.1–1.4)
	50–80 min	0.6 (0.5–0.7)	0.5 (0.5–0.6)
	80–∞ min	2.8 (2.4–3.2)	2.5 (2.3–2.7)
Mean blood sampling time	0–55 min	1.5 (1.3–1.8)	1.3 (1.2–1.5)
	55–100 min	0.8 (0.7–1.0)	0.7 (0.7–0.8)
	100–∞ min	2.4 (2.1–2.8)	2.1 (2.0–2.4)
Maximum blood sampling time	0–60 min	1.6 (1.4–1.9)	1.4 (1.3–1.6)
	60–120 min	1.0 (0.9–1.2)	0.9 (0.8–1.0)
	120–∞ min	2.1 (1.8–2.5)	1.9 (1.7–2.1)
Effective dose from FDG-PET/CT (mSv)			
CT dose only	—	3.4 (3.0–3.9)	9.2 (7.6–11.3)
FDG + CT (minimum sampling time)	50–80 min + CT	4.0 (3.7–4.5)	9.7 (8.2–11.9)
	0–80 min + CT	5.4 (5.1–5.9)	11.0 (9.6–13.2)
FDG + CT (mean sampling time)	55–100 min + CT	4.1 (3.9–4.7)	9.9 (8.5–12.1)
	0–100 min + CT	5.7 (5.4–6.2)	11.3 (10.0–13.6)

FDG + CT (maximum sampling time)	60–120 min + CT	4.3 (4.1–4.9)	10.1 (8.7–12.3)
	0–120 min + CT	6.0 (5.6–6.5)	11.5 (10.2–13.8)

Data are presented as median (IQR).

Supplementary Table S4. Median and interquartile range (IQR) of CAs at each sampling point

	Center RD	Center SD	*p*-value
"pre"	8.5	8.3	0.31
	(6.4–15.4)	(5.3–12.5)	
"+FDG"	9.8	7.8	0.69
	(5.6–14.7)	(5.0–16.7)	
"+FDG+CT"	8.8	11.0	0.27
	(5.8–13.8)	(5.4–15.5)	

Center RD: reduced-dose PET/CT protocol; Center SD: standard-dose PET/CT protocol
Data are presented as median (IQR) per 1,000 cells.
p-values were calculated using Mann–Whitney *U* test.

Supplementary Table S5. Median and IQR of ΔCAs per 1,000 cells between sampling points

	Center RD	*p*-value	Center SD	*p*-value
"+FDG" − "pre"	0.0 (−4.0 to 3.3)	–	0.0 (−3.7 to 5.3)	0.39
"+FDG+CT" − "+FDG"	0.3 (−3.7 to 3.3)	–	1.7 (−2.5 to 5.2)	0.23
"+FDG+CT" − "pre"	−0.4 (−3.5 to 2.5)	–	1.2 (−1.5 to 6.6)	**0.016**

Center RD: reduced-dose PET/CT protocol; Center SD: standard-dose PET/CT protocol
Data are presented as median (IQR) per 1,000 cells.
Friedman test showed no significant temporal change in Center RD ($p = 0.87$), whereas a significant temporal change was observed in Center SD ($p = 0.023$).
Pairwise comparisons were not performed when the Friedman test was not significant.
In Fig. 2b, "ns" for Center RD denotes a non-significant overall Friedman test, not pairwise comparison results.
Pairwise *p*-values are shown only for groups with a significant Friedman test and were calculated using Wilcoxon signed-rank tests with Holm adjustment.

Supplementary Table S6. Model fit diagnostics for the primary Poisson generalized linear mixed model

Diagnostic	Value	Interpretation
Model convergence	No convergence warning	Acceptable
Singular fit	No	No evidence of singular fit, stable estimation
Overdispersion	Dispersion ratio = 0.89<br>$p = 0.94$	No evidence of overdispersion
Simulation-based dispersion test	Dispersion =0.99<br>$p = 0.83$	No evidence of dispersion
Simulation-based zero-inflation test	Ratio = 0.90<br>$p = 1.00$	No evidence of zero inflation

Simulation-based dispersion and zero-inflation tests were performed using the DHARMa package in R.

Supplementary Table S7. Sensitivity Poisson generalized linear mixed model excluding the interaction term between FDG effective dose and CT effective dose

Complete-case GLMM (n = 151 patients, 453 observations)				
	Rate ratio	95% CI	*p*-value	
Center	1.00	0.82–1.22	1.00	
SD vs RD				
Sex	1.07	0.86–1.34	0.56	
Male vs Female				
Age (per 10 years)	1.10	1.00–1.21	**0.043**	*
Smoking	1.13	0.90–1.41	0.29	
Ever vs never				
Chemotherapy history	2.03	1.60–2.58	**< 0.001**	***
Yes vs no				
FDG effective dose (per 1 mSv)	1.02	0.97–1.08	0.37	
CT effective dose (per 1 mSv)	1.01	1.00–1.02	**0.010**	*

RD: reduced-dose, SD: standard-dose, CI: confidence interval; CA: chromosomal aberration; GLMM: generalized linear mixed-effects model

The primary GLMM was performed as a complete-case analysis and included 151 CA-evaluable patients: Center RD, n = 101; Center SD, n = 50.

Five CA-evaluable patients at Center SD were excluded because of missing smoking-history data.

The chemotherapy-naïve analysis was derived from this complete-case population by excluding patients with chemotherapy history.

Rate ratios for age, FDG effective dose, and CT effective dose are expressed per 10-year, 1-mSv, and 1-mSv increase, respectively.

Supplementary Table S8. Sensitivity negative binomial generalized linear mixed model for factors associated with CA frequency

Complete-case GLMM (n = 151 patients, 453 observations)				
	Rate ratio	95% CI	*p*-value	
Center				
SD vs RD	0.99	0.81–1.21	0.93	
Sex				
Male vs Female	1.07	0.85–1.33	0.58	
Age (per 10 years)	1.10	1.00–1.21	**0.042**	*
Smoking				
Ever vs never	1.13	0.90–1.41	0.28	
Chemotherapy history				
Yes vs no	2.04	1.60–2.59	**< 0.001**	***
FDG effective dose (per 1 mSv)	0.94	0.77–1.15	0.54	
CT effective dose (per 1 mSv)	1.01	1.00–1.02	**0.015**	*
Interaction: FDG effective dose × CT effective dose (per 1 mSv × 1 mSv)	0.96	0.87–1.06	0.41	
Chemotherapy-naïve complete-case GLMM (n = 125 patients, 375 observations)				
	Rate ratio	95% CI	*p*-value	
Center				
SD vs RD	0.92	0.73–1.15	0.44	
Sex				
Male vs Female	0.96	0.75–1.22	0.73	
Age (per 10 years)	1.11	1.01–1.22	**0.031**	*
Smoking				
Ever vs never	1.30	1.02–1.64	**0.032**	*
FDG effective dose (per 1 mSv)	0.98	0.78–1.24	0.90	
CT effective dose (per 1 mSv)	1.01	1.00–1.02	**0.031**	*
Interaction: FDG effective dose × CT effective dose (per 1 mSv × 1 mSv)	0.98	0.88–1.10	0.77	

RD: reduced-dose, SD: standard-dose, CI: confidence interval; CA: chromosomal aberration; GLMM: generalized linear mixed-effects model

The primary GLMM was performed as a complete-case analysis and included 151 CA-evaluable patients: Center RD, n = 101; Center SD, n = 50.

Five CA-evaluable patients at Center SD were excluded because of missing smoking-history data.

The chemotherapy-naïve analysis was derived from this complete-case population by excluding patients with chemotherapy history.

Detailed analytic sets are shown in Supplementary Table S2.

Rate ratios for age, FDG effective dose, and CT effective dose are expressed per 10-year, 1-mSv, and 1-mSv increase, respectively.

Supplementary Table S9. Association of time-window-based FDG dose calculations with ΔCAs per 1,000 cells ("+FDG" − "pre")

Dose calculation	Median dose (mSv) (IQR)	Linear regression (*β*) (95% CI)	*p*-value	Spearman's correlation *ρ*	Spearman *p*-value	R^2
0–50 min integration	1.3 (1.2 to 1.5)	0.34 (−4.03 to 4.71)	0.87	0.04	0.60	< 0.001
0–55 min integration	1.5 (1.3 to 1.7)	0.32 (–3.69 to 4.32)	0.88	0.04	0.60	< 0.001
0–60 min integration	1.6 (1.4 to 1.8)	0.31 (−3.45 to 4.07)	0.88	0.04	0.59	< 0.001
0–∞ min integration	4.5 (4.0 to 5.2)	0.10 (−1.18 to 1.38)	0.88	0.04	0.59	< 0.001

FDG dose was calculated using the lower bound, upper bound, or without timing correction based on the clinical blood sampling window.
β represents the change in ΔCAs per 1,000 cells per 1-mSv increase in dose.
All models were based on the same set of patients with available PNA-FISH data (n = 156).
Spearman correlation coefficients are provided as nonparametric sensitivity analyses.

Supplementary Table S10. Association of time-window-based FDG dose calculations (FDG+CT) with ΔCAs per 1,000 cells ("+FDG+CT" − "+FDG")

Dose calculation	Median dose (mSv) (IQR)	Linear regression (β) (95% CI)	p-value	Spearman's correlation ρ	Spearman p-value	R^2
FDG (50–80 min) + CT	4.5 (3.8 to 8.6)	0.28 (0.01 to 0.55)	0.039	0.14	0.08	0.03
FDG (55–100 min) + CT	4.8 (4.0 to 8.8)	0.28 (0.01 to 0.55)	0.040	0.14	0.07	0.03
FDG (60–120 min) + CT	4.9 (4.2 to 8.9)	0.28 (0.01 to 0.55)	0.040	0.14	0.07	0.03
CT dose only	3.9 (3.2 to 8.0)	0.29 (0.02 to 0.55)	0.037	0.14	0.08	0.03

FDG dose was calculated using the lower bound, upper bound, or without timing correction based on the clinical blood sampling window.

β represents the change in ΔCAs per 1,000 cells per 1-mSv increase in dose.

All models were based on the same set of patients with available PNA-FISH data (n = 156).

Spearman correlation coefficients are provided as nonparametric sensitivity analyses.

Supplementary Table S11. Association of time-window-based FDG dose calculations (FDG+CT) with ΔCAs per 1,000 cells ("+FDG+CT" − "pre")

FDG dose range	Median dose (mSv) (IQR)	Linear regression (β) (95% CI)	p-value	Spearman's correlation ρ	Spearman p-value	R^2
FDG (0–80 min) + CT	5.9 (5.2 to 9.7)	0.51 (0.25 to 0.78)	< 0.001	0.25	< 0.01	0.09
FDG (0–100 min) + CT	6.3 (5.6 to 10.0)	0.51 (0.25 to 0.78)	< 0.001	0.25	< 0.01	0.09
FDG (0–120 min) + CT	6.6 (5.9 to 10.3)	0.51 (0.25 to 0.78)	< 0.001	0.25	< 0.01	0.09
FDG (0–∞) + CT	8.9 (7.9 to 12.3)	0.50 (0.24 to 0.77)	< 0.001	0.25	< 0.01	0.08

FDG dose was calculated using the lower bound, upper bound, or without timing correction based on the clinical blood sampling window.

β represents the change in ΔCAs per 1,000 cells per 1-mSv increase in dose.

All models were based on the same set of patients with available PNA-FISH data (n = 156).

Spearman correlation coefficients are provided as nonparametric sensitivity analyses.

Supplementary Table S12. Median values and IQR for each group in each center

	Center RD			Center SD		
	Chemo -	Chemo +	*p*-value	Chemo -	Chemo +	*p*-value
"pre"	8.0 (6.1–13.8)	15.4 (7.6–23.5)	0.028	7.1 (3.8–10.6)	20.1 (14.4–26.8)	< 0.001
"+FDG"	9.1 (5.1–13.9)	13.7 (9.8–18.3)	0.014	7.4 (4.4–11.6)	23.8 (6.2–36.7)	0.004
"+FDG+CT"	7.7 (5.4–12.6)	15.9 (12.1–19.3)	< 0.001	9.6 (4.3–13.2)	17.6 (15.1–31.4)	< 0.001

Center RD: reduced-dose PET/CT protocol; Center SD: standard-dose PET/CT protocol

Data are presented as median (IQR) per 1,000 cells.

p-values were calculated using Mann–Whitney *U* test.

Supplementary Table S13. Median and IQR of ΔCAs for each group in each center

	Center RD				Center SD			
	Chemo −	*p*-value	Chemo +	*p*-value	Chemo −	*p*-value	Chemo +	*p*-value
"+FDG" − "pre"	0.0 (−3.8 to 2.9)	–	−1.3 (−8.0 to 5.9)	–	0.0 (−2.1 to 4.6)	0.40	−3.0 (−5.8 to 10.8)	–
"+FDG+CT" − "+FDG"	−0.1 (−3.7 to 2.3)	–	1.4 (−4.4 to 4.2)	–	1.7 (−2.5 to 4.1)	0.40	3.6 (−3.5 to 8.7)	–
"+FDG+CT" − "pre"	−1.2 (−3.4 to 2.4)	–	0.6 (−6.1 to 4.9)	–	1.2 (−0.9 to 5.8)	**0.017**	2.0 (−2.6 to 8.9)	–

Center RD: reduced-dose PET/CT protocol; Center SD: standard-dose PET/CT protocol

Data are presented as median (IQR) per 1,000 cells.

Friedman test showed a significant time-dependent change only in Center SD Chemo− patients ($p = 0.037$).

Pairwise comparisons were not performed when the Friedman test was not significant.

Pairwise p-values are shown only for groups with a significant Friedman test and were calculated using Wilcoxon signed-rank tests with Holm adjustment.
